# Proposed development of the Criminal Justice Translation and Clinical Science (CJ-TRACS) network

**DOI:** 10.1017/cts.2023.531

**Published:** 2023-04-17

**Authors:** Nickolas D. Zaller, Michele Staton, Margaret M. Gorvine, Martha Tillson, Jason Glenn, George Pro, Carrie Oser, Megha Ramaswamy

**Affiliations:** 1 Southern Public Health and Criminal Justice Research Center, University of Arkansas for Medical Sciences Fay W. Boozman College of Public Health, Little Rock, AR, USA; 2 Department of Behavioral Science, College of Medicine, University of Kentucky, Lexington, KY, USA; 3 Department of Population Health, School of Medicine, Kansas University, Kansas City, KS, USA; 4 Department of Sociology, College of Arts and Sciences, University of Kentucky, Lexington, KY, USA

**Keywords:** Criminal justice system, incarceration, translational science, health disparities, research network

## Why a CTSA Criminal Justice Research Network is Needed

Criminal Justice (CJ) settings, that is jails, prisons, and community correctional settings such as probation and parole, provide ample opportunities to engage difficult-to-reach populations who have significant underlying health conditions, including a high burden of chronic [1] and infectious diseases [2–3] and behavioral health disorders [4–5]. Due to high-risk lifestyles associated with untreated mental health disorders, adverse childhood experiences (ACEs), structural violence, substance use, and crime, as well as challenges associated with poverty and limited health care, a high percentage of CJ-involved individuals have significant, untreated health needs [6–7]. By CJ-involved, we mean individuals who are either incarcerated in prisons or jails or individuals who have experienced incarceration but who currently reside in the community. However, public health interventions are under-utilized both within the context of CJ settings and among individuals involved in the CJ system, including those re-entering communities from institutional settings.

Additionally, incarceration rates are disproportionately high among people of color as a result of the discriminatory war on drugs and other punitive policies. This systemic discrimination creates a nexus of intersectional burden for communities of color that are simultaneously tasked with supporting and caring for individuals in re-entry [8–9]. Racial health disparities are further perpetuated.

While 20 million Americans are currently incarcerated or have been in the past, only a small fraction (∼0.1%) of NIH grants focus on CJ-related research [10].

Compounding the under-investment in CJ-related research, CJ settings can be overlooked for medical and public health-related research partly because of the unique challenges that correctional facilities (both jails and prisons) pose to conducting research-related activities, but also related to the lack of preparedness and infrastructure for people who want to do this research. Additionally, the history of medical exploitation among individuals involved in the CJ system has necessitated more rigorous ethical oversight among research participants within this system [11–12]. Partnerships between correctional systems and academic medical systems can provide research and clinical training opportunities for an array of healthcare specialties. A more robust healthcare system within correctional environments may yield important opportunities to engage incarcerated patients in research studies, including clinical trials, behavioral health interventions, and/or linkage interventions designed to facilitate continuity of care between correctional and community-based healthcare providers. The results of studies conducted within correctional environments translate to a more skilled and empowered healthcare workforce that can provide people involved in the CJ system more informed care both within the carceral system, as well as linked to and within community settings.

To address the substantial health disparities among CJ-involved populations, there must be more investment in translational science involving individuals impacted by incarceration. Jennifer Alvidrez and Nathan Stinson at the National Institute on Minority Health and Health Disparities developed the translational health disparities continuum to advance health disparities research beyond translational stages T0 (identification), T1 (development), and T2 (testing intervention effectiveness) and move towards T3 (implementation) and T4 (dissemination) so that the benefits of translational research can be realized to combat racial health disparities [13]. There remains a pressing need to apply these translational stages to issues around incarceration and health, including engaging the community at every stage of the translational framework. The National Center for Advancing Translation Sciences offers resources to research hubs through the Clinical and Translational Science Awards (CTSA) with the intent to catalyze collaborations to speed the delivery of evidence-based findings to patients.

## Laying the Foundation for a Criminal Justice Translational and Clinical Science (CJTRACS) Network

In response to this need, we are working toward the development of the Criminal Justice Translational and Clinical Science (CJTRACS) Network (www.kumc.edu/cjtracs) in order to support translational science across the criminal justice continuum. Initially supported through a CTSA pilot grant to three institutions (University of Arkansas for Medical Sciences; UAMS; University of Kentucky; UKY; and Kansas University Medical Center; KUMC), the primary goal of the CJTRACS Network is to develop a national CTSA research network to support translational science across the CJ continuum. An ethically sound and structurally competent research agenda is critical to advancing public health among CJ populations and communities that are disproportionately burdened by health disparities and mass incarceration (i.e., racial, sexual, and gender minority populations, rural populations and populations with behavioral health needs). In this editorial, we outline our vision for a network of researchers dedicated to translational and clinical science across the criminal justice continuum. Such a network would build upon existing infrastructure at CTSA-affiliated institutions by expanding capacity to address the unique ethical challenges of conducting research among carceral populations, provide training and educational opportunities to investigators to conduct rigorous CJ-related research, and leverage existing CTSA-funded mechanisms, e.g. pilot awards and the KL2 training program, to support innovative research among CJ-impacted populations.

## Leveraging Existing CTSA Resources to Further Develop and Expand Criminal Justice Research

The pilot project CTSAs all offer training opportunities and support for scholars from underrepresented groups as well as in health disparities research career development. UAMS’ programs include the *Mini Grants for Under-Represented Faculty Researchers* program, which supports mini-grants among UAMS under-represented faculty proposing health disparities research. UKY’s CTSA includes an *Integrated Special Populations Core*, which aims to increase the presence of underrepresented populations within the research workforce as well as study participants. As part of this core, UKY offers the *Disparities Researchers Equalizing Access for Minorities (DREAM) Scholars Program*, which provides mentored health equity research training for exceptional underrepresented minority scholars at the predoctoral, postdoctoral, and assistant professor levels. Another opportunity to establish a racial health equity workforce pipeline is through UKY’s *Students Participating as Ambassadors for Research in Kentucky (SPARK)* program. SPARK provides undergraduate students from underrepresented backgrounds opportunities to take intensive short courses on Health Equity and Research Methods while engaging in book discussions, professional presentations, and conducting research in their home communities under the mentorship of a faculty and community member. The Kansas University Medical Center extends training through the *TL1 Trainee Program*, which targets pre- and postdoctoral students to provide trainees with the skills, confidence, motivation, and enhanced career trajectory to move to the next stage of their translational research career. All of these training programs are potential “homes” for specialized CJ-related research training.

Extending the community-engagement lens to include people who were previously incarcerated in outreach and training, as well as community members affected by mass incarceration, could be incorporated into existing training programs as well. For example, UAMS has the *Community-Based Participatory Research (CBPR) Scholars Program* which seeks to increase community-partnered research at UAMS to better serve the research needs of the Arkansas community and to reduce health disparities. UAMS also has the *Translational Research Innovations and Partners (TRIP) Program* which provides research support to first- or second-year medical students who are interested in learning about and participating in community-based research focused on medically underserved, rural, and minority populations, including CJ populations. Through a CTSA Community Engagement & Research Core, UKY has two *Rural Research Hubs*, which can be a resource for community, clinical, and academic partners engaged in community-engaged research to connect with CJ agencies in rural areas. Finally, KU has the *Trailblazer Awards*, which fund the development of community-engaged research or engage special populations in clinical and translational research, which could include an additional emphasis on CJ-related research.

Each institution has unique programing to meet translational research directives which could be directly applied to CJ-related research. The UAMS Translational Research Institute offers numerous training and research development programs focused on training a diverse research workforce to address key scientific challenges related to health disparities. In addition to the aforementioned, these include the *UAMS Research Academy*, which seeks to build a sustainable research community through creating innovative care models and transformative breakthroughs in medicine; the *Community Partners Educated as Arkansas Research Leaders (CPEARL) Program*, which is a 6-month leadership development training program targeting leaders and emerging leaders within nonprofit community-based organizations located in Arkansas; and the *Implementation Sciences Scholars Program*, which provides didactive and research support to UAMS faculty seeking to learn how to implement new practice guidelines and/or other implementation or deimplementation approaches that will improve medical care.

The UKY CTSA also supports the *Professional Student Mentored Research Fellowship Program (PSMRF),* which provides individually tailored mentored research fellowships in clinical and translational science for professional students (e.g. MD, DMD, PharmD, Public Health, Clinical Psychology, Nursing DNP, and Physician Assistant’s Program).

The KUMC CTSA, *Frontiers*, addresses the science and healthcare workforce needs for clinical and translational research (CTR) in the Midwest. Alumni of Frontiers career development programs are not only emerging as regional and national leaders in research, but they also drive research that sets the foundation for improvements in clinical and public health practice – advancing the delivery of innovations to the patients and communities that need them. Collectively, the *Frontiers* programs bring together more than 200 federally funded investigators in CTR drawn from eight regional Midwestern institutions with strong track records of working across state lines and institutional boundaries to advance interdisciplinary research.

These are just some of the existing programs that each of these institutions support, in addition to a KL2 Scholars program offered by each institution which fosters career development to ensure a firm grounding in CTR competencies and preparation to conduct rigorous and reproducible research. These programs, while not specific to CJ-related research, can be leveraged to support a program of CJ-related research at each institution and at other CTSAs nationally. Harnessing existing research mechanisms as resources for translational researchers interested in CJ and health will help support a more robust program of health disparities research among CJ-impacted populations. Much of the work originating from these centers and institutes could form the basis of future NIH grants geared specifically towards criminal justice populations using a translational science lens. A growing network of CJ scholars will expand the reach of these organizations through inter-institutional partnerships, open communication platforms about mutual interests and emerging research topics, and continued engagement with community members most affected by mass incarceration.
